# Diffusion-driven fed-batch fermentation in perforated ring flasks

**DOI:** 10.1007/s10529-024-03493-0

**Published:** 2024-05-17

**Authors:** Clara Lüchtrath, Felix Lamping, Sven Hansen, Maurice Finger, Jørgen Magnus, Jochen Büchs

**Affiliations:** 1 AVT-Biochemical Engineering, RWTH Aachen University, Forckenbeckstraße 51, 52074 Aachen, Germany; 2 Evonik Operations GmbH, Paul-Baumann-Straße 1, 45772 Marl, Germany

**Keywords:** Additive manufacturing, Fed-batch, Oxygen transfer rate, Respiration activity monitoring system, Ring flask

## Abstract

**Purpose:**

Simultaneous membrane-based feeding and monitoring of the oxygen transfer rate shall be introduced to the newly established perforated ring flask, which consists of a cylindrical glass flask with an additional perforated inner glass ring, for rapid bioprocess development.

**Methods:**

A 3D-printed adapter was constructed to enable monitoring of the oxygen transfer rate in the perforated ring flasks. *Escherichia coli* experiments in batch were performed to validate the adapter. Fed-batch experiments with different diffusion rates and feed solutions were performed.

**Results:**

The adapter and the performed experiments allowed a direct comparison of the perforated ring flasks with Erlenmeyer flasks. In batch cultivations, maximum oxygen transfer capacities of 80 mmol L^−1^ h^−1^ were reached with perforated ring flasks, corresponding to a 3.5 times higher capacity than in Erlenmeyer flasks. Fed-batch experiments with a feed reservoir concentration of 500 g glucose L^−1^ were successfully conducted. Based on the oxygen transfer rate, an ammonium limitation could be observed. By adding 40 g ammonium sulfate L^−1^ to the feed reservoir, the limitation could be prevented.

**Conclusion:**

The membrane-based feeding, an online monitoring technique, and the perforated ring flask were successfully combined and offer a new and promising tool for screening and process development in biotechnology.

**Supplementary Information:**

The online version contains supplementary material available at 10.1007/s10529-024-03493-0.

## Introduction

Shake flasks are widely used in biotechnology for strain screening, media development, and early-stage process development (van Suijdam et al. 1978; Büchs 2001; Takahashi and Aoyagi 2018). Shake flasks are, compared to stirred tank reactors, easy to handle, require little material, and can simply be parallelized so that many experiments can be performed simultaneously (van Suijdam et al. 1978; Verglio et al. 1998; Büchs 2001; Takahashi and Aoyagi 2020). However, the gas/liquid mass transfer is limited in shake flasks, which results in lower maximum oxygen transfer capacities compared to stirred tank reactors (McDaniel et al. 1965; van Suijdam et al. 1978). However, for aerobic cultivation, the prevention of oxygen limitation is of utmost importance (Hansen et al. 2022). The key parameter for the oxygen supply is the gas/liquid mass transfer area (Büchs 2001). The area is dependent on a thin liquid film, which is formed on the hydrophilic flask wall during shaken cultivation (van Suijdam et al. 1978). The film is influenced by the viscosity (η) of the bulk liquid, the shaking frequency (n), the shaking diameter (d_0_), the filling volume (V_L_), and the shape of the flask (Maier and Büchs 2001). One possible modification of the latter is a baffled cylindrical flask, as investigated by Takahashi and Aoyagi (2020). An additional modification is the perforated ring flask, as introduced by Hansen et al. (2022). This cylindrical flask contains an internal, concentric ring, resulting in two compartments. To allow mixing between these two compartments, two perforations are present at the bottom of the inner ring. Thus, the gas/liquid mass transfer area is drastically increased, as not only one wall is wetted with a thin liquid film but three. As a result, oxygen transfer rates (OTR) of 140 mmol L^−1^ h^−1^ were observed under practical operating conditions. These high oxygen transfer rates are close to rates reached in stirred tank reactors and can be achieved without losing the mentioned advantages of shake flasks (Hansen et al. 2022).

Another important factor for screening and process development in shake flasks is the introduction of fed-batch techniques since industrial processes are often operated in a fed-batch mode (Panula-Perälä et al. 2008; Toeroek et al. 2015; Bolmanis et al. 2023). Overflow metabolism, oxygen limitation, and undesirable by-product formation can be avoided (Jeude et al. 2006; Bolmanis et al. 2023). Various techniques for small-scale fed-batch cultivations in shake flasks are available. The Enpresso System (Enpresso GmbH, Berlin, Germany) enables fed-batch conditions by enzymatic degradation of polysaccharides (Panula-Perälä et al. 2008). For fed-batch cultivations also “Feed beads” are available (Kuhner Shaker GmbH, Herzogenrath, Germany), where glucose is embedded into a silicone elastomer and is released upon contact with the culture broth (Jeude et al. 2006). Another technology is the membrane-based fed-batch shake flask. A membrane separates a feed solution from the culture broth. Across this membrane, a concentration gradient is present since the concentration of the reservoir feed solution is higher than that of the culture broth. The concentration gradient acts as a driving force for a continuous diffusion-driven substrate delivery during cultivation (Bähr et al. 2012; Philip et al. 2017; Habicher et al. 2019).

**Fig. 1:**
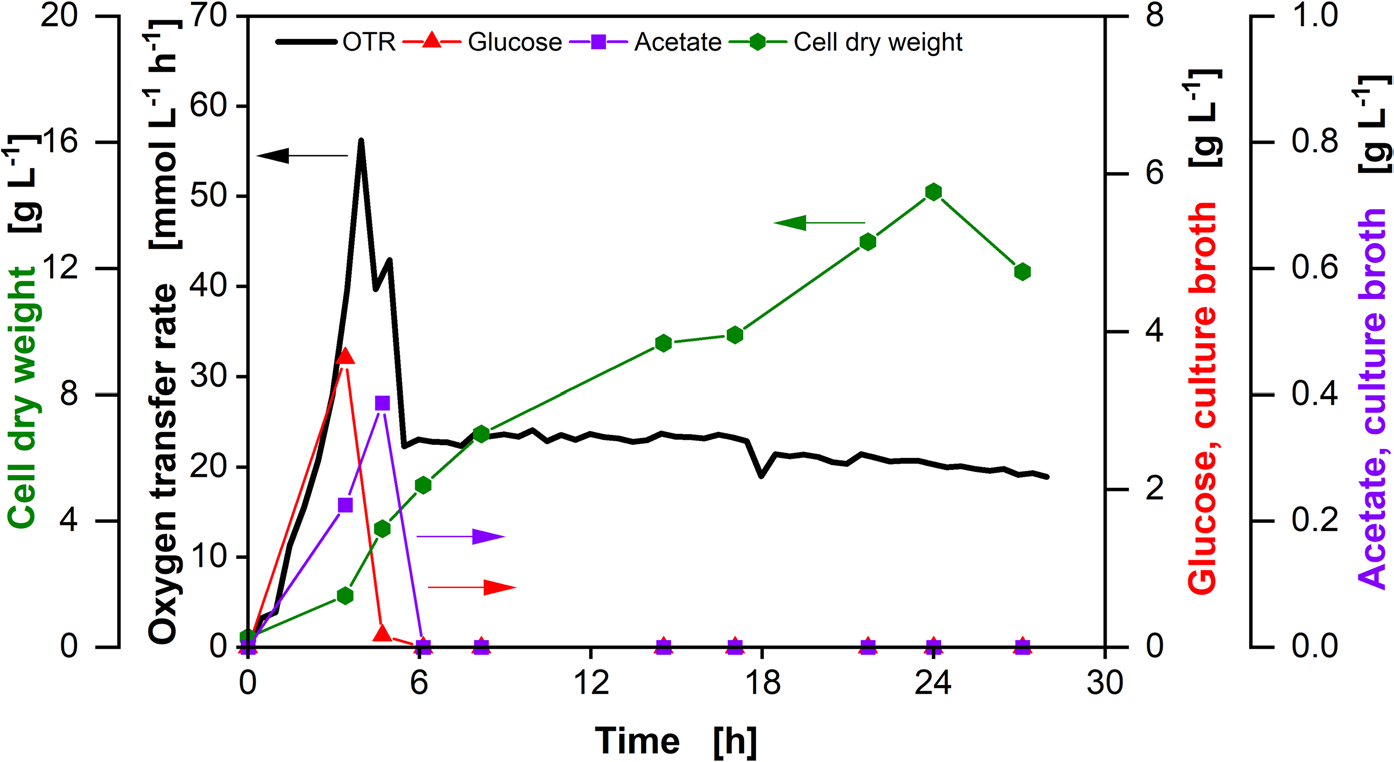
Fed-batch fermentation of *E. coli* BL21 (DE3) pRhotHi-2-EcFbFP in the membrane-based fed-batch shake flask. The oxygen transfer rate (black line) was monitored via a RAMOS device. The glucose (red triangles) and acetate (purple squares) concentration in the culture broth and the cell dry weight (green diamonds) were analyzed. Cultivation conditions: 250 mL shake flask, V_L_ = 10 mL, d_0_ = 50 mm, n = 350 rpm, T = 37 °C, initial optical density OD_600_ = 0.7, V_Reservoir_ = 2 mL, c_Reservoirs_ = 250 g L^−1^, 200 mM MOPS buffer (adapted from Philip et al. 2017)

To ensure consistency of cultivation conditions during initial process development and to avoid complications during the subsequent scale up, online monitoring techniques like the Respiration Activity Monitoring System (RAMOS) are crucial for early process development (Anderlei and Büchs 2001; Wewetzer et al. 2015). With the RAMOS device, the physiological state of aerobic cultivations representing phenomena like oxygen limitation, diauxic growth or substrate limitation can be recognized (Anderlei and Büchs 2001; Anderlei et al. 2004). A striking advantage of this techniques is the high level of information content it provides without the need for sampling or complex offline analysis (Anderlei et al. 2004; Klöckner and Büchs 2011; Takahashi and Aoyagi 2020). In previous studies, the applicability of the RAMOS device in combination with the membrane-based fed-batch shake flask system was investigated in detail (Bähr et al. 2012; Philip et al. 2017, 2018; Habicher et al. 2019). Philip et al. (2017) presented data on the change of the glucose and the acetate concentrations in the culture broth and the cell growth over a fed-batch cultivation in a membrane-based fed-batch shake flask (Fig. 1).

In this study, a newly designed perforated ring flask, called large perforated ring flask, is introduced, which has a greater diameter than the one introduced by Hansen et al. (2022), in this study called small perforated ring flask. The newly designed large perforated ring flask is combined with the membrane-based fed-batch system, to gain a shaken bioreactor enabling conditions much closer to stirred tank reactors.

## Materials and methods

### Respiration activity monitoring system

To determine the oxygen transfer rate in large perforated ring flasks, the respiration activity monitoring system (RAMOS) was used. It enables a non-invasive, online monitoring of the respiratory activity of microorganisms (Anderlei and Büchs 2001; Anderlei et al. 2004). An in-house built device was used in this work. A commercial version is available from HiTec Zang (Herzogenrath, Germany). From Kuhner AG (Birsfelden, Swiss) a modified version became recently available.

### Perforated ring flask

**Fig. 2:**
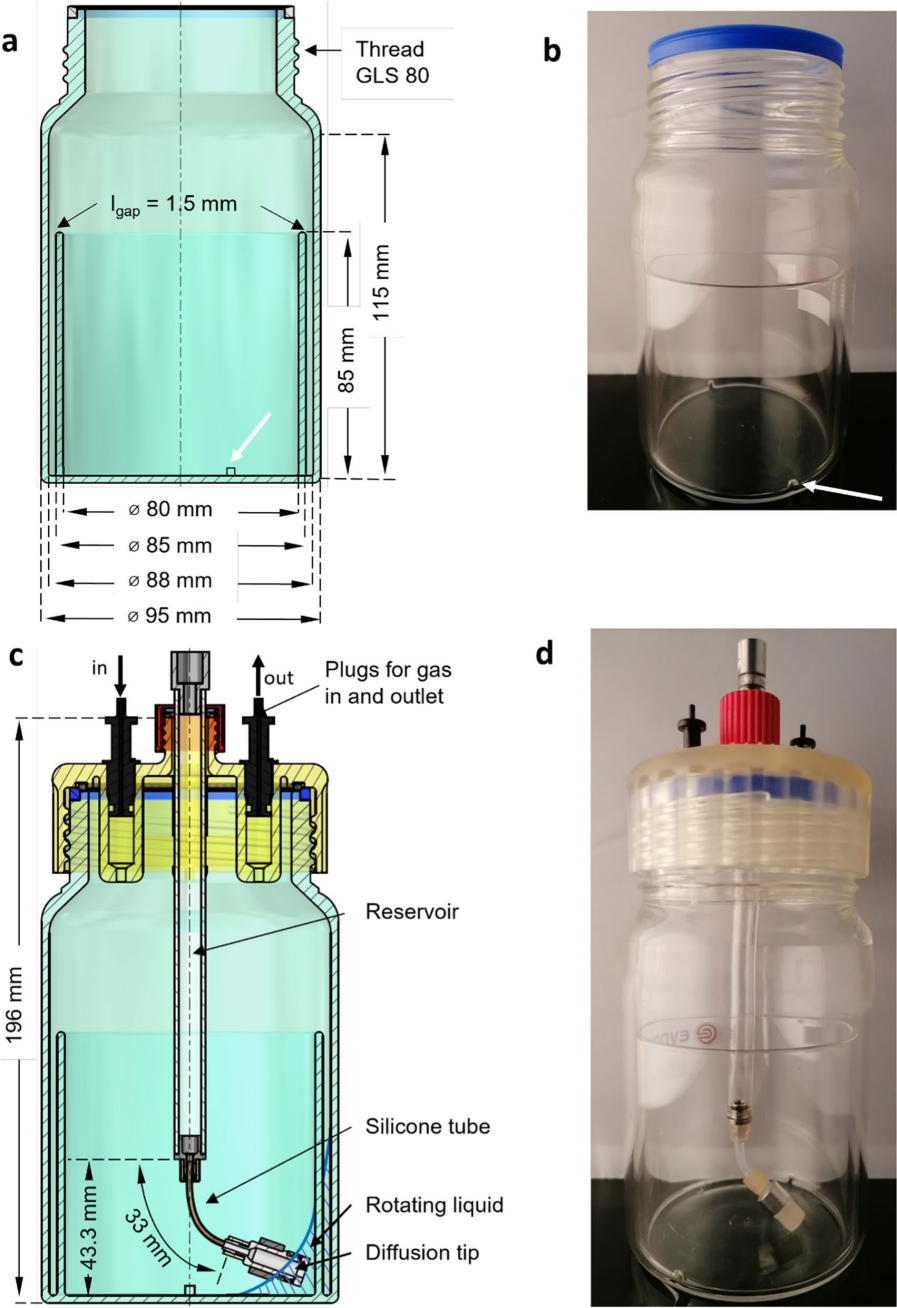
Design and structure of the large perforated ring flask. Dimensions (**a**) and picture (**b**) of an empty large perforated ring flask. Dimensions (**c**) and picture (**d**) of a large perforated ring flask with a 3D printed adapter for connecting to a RAMOS device. To the adapter, a reservoir for fed-batch cultivation is mounted (Fig. S4a). Plugs for air inlet and exhaust gas outlet are inserted into the adapter (**c**, **d**). The white arrow in (a) + (b) point at one of the two perforations (3 × 3 mm)

The perforated ring flask was introduced by Hansen et al. (2022). It consists of a cylindrical vessel, with a concentric ring attached to the bottom (Fig. S1). The ring divides the flask into two compartments. Two perforations connect the compartments and enable the liquid to distribute between the inner and the outer compartment. In this work, a newly designed large perforated ring flask is introduced (Fig. 2).

### Preparation of membrane-based fed-batch shake flasks

The preparation of the membrane-based fed-batch shake flask was conducted according to Philip et al. (2017) and Habicher et al. (2019). In this study, a regenerated cellulose membrane (RCT-NatureFlex NP, Reichelt Chemietechnik GmbH Co., Heidelberg, Germany) with a thickness of 42 µm and a molecular weight cut‐off of 10–20 kDa was applied. For diffusion tips with a diffusion area of A = 18.1 mm^2^ (d_tip1_ = 4.8 mm), membrane discs of 16 mm, for diffusion tips with a diffusion area of A = 54.1 mm^2^ (d_tip2_ = 8.3 mm), membrane discs of 24 mm were punched out of a larger piece of membrane (Fig. S4b, c). To fix the membrane on the tip, a flexible and biocompatible silicone tube (Saint Gobain, Versilic® Silicone Tubing Article numbers 760330 and 760590) is dragged over the membrane on the diffusion tip using an in-house built apparatus (Fig S4d). The diffusion tip was filled with 200 µL for the diffusion tips with A = 18.1 mm^2^ or 900 µL for the diffusion tips with A = 54.1 mm^2^ of deionized water. The test for possible leakage presented by Philip et al. (2017) was omitted, since the assembly of the diffusion tip was optimized and leakage was never observed (Habicher et al. 2019). Subsequently, the reservoir (Fig. 2c, Fig. S4a) was assembled. A flexible tube, which enables the rotation of the diffusion tip in the liquid, consists of biocompatible silicone (for diffusion tips with A = 18.1 mm^2^: 2 × 4 mm silicone tubing, for diffusion tips with A = 54.1 mm^2^: 3 × 5 mm silicone tubing, VWR International GmbH, Darmstadt, Germany). After assembling, the reservoir was autoclaved at 121 °C for 20 min.

### Media

For pre-and main cultivations of *Escherichia coli* BL21, a modified Wilms-MOPS medium (Wilms et al. 2001; Mühlmann et al. 2018) was applied. If not stated otherwise, the chemicals were purchased from Carl Roth GmbH & Co. KG (Karlsruhe, Germany). For 1 L medium, the following components were used: 6.98 g (NH_4_)_2_SO_4_, 3 g K_2_HPO_4_, 2 g Na_2_SO_4_ (Merck KGaA, Darmstadt, Germany), 41.85 g 3-(N-morpholino)-propanesulfonic acid (200 mM MOPS), 0.5 g MgSO_4_·7 H_2_O, 0.01 g thiamine-hydrochloride, 1 mL trace elements solution. The trace element solution consisted of 0.54 g ZnSO_4_·7 H_2_O (Merck KGaA, Darmstadt, Germany), 0.48 g CuSO_4_·5 H_2_O (Merck KGaA, Darmstadt, Germany), 0.3 g MnSO_4_·H_2_O, 0.54 g CoCl_2_·6 H_2_O, 41.76 g FeCl_3_·6 H_2_O, 1.98 g CaCl_2_·2 H_2_O, 33.39 g Na_2_EDTA·2 H_2_O per liter. Ampicillin (Sigma Aldrich Chemie GmbH, Steinheim, Germany) was supplemented to the medium to reach a concentration of 0.1 g L^−1^. The pH value was adjusted to 7.5 with 5 M NaOH. For pre-cultivation, 10 g glucose L^−1^, and for batch main cultures 20 g glucose L^−1^ was supplemented. If not stated otherwise, no glucose was initially supplemented for fed-batch main cultivations. For cultivations with additional ammonium feed, 40 g ammonium L^−1^ in form of ammonium sulfate was dissolved within the glucose feed solution. The solution with additional ammonium was sterile filtered (pore diameter d = 0.22 µm, Merck KGaA, Darmstadt, Germany), whereas the glucose solution without ammonium was autoclaved before it was used in cultivations.

### Cultivation conditions

Cryo-cultures of *E. coli* were cultivated in Wilms-MOPS medium and were stored with 15% glycerol at − 80 °C. Online monitored pre-cultures were inoculated with 1 µL cryo- culture per mL and were cultivated overnight in 250 mL shake flasks with a filling volume V_L_ of 10 mL at a temperature T of 30 °C, with a shaking diameter d_0_ of 50 mm and a shaking frequency n of 350 rpm. The main culture was cultivated in the large perforated ring flask (Fig. 2) with a V_L_ of 25 mL at a T of 37 °C, with a d_0_ of 50 mm and n of 250 rpm. Each main culture was conducted in replicates inoculated with the same pre-culture. The number of performed replicates is specified (with the symbol “n”) for each cultivation condition.

### Measurement of the optical density

The optical density at the beginning and the end of the cultivation (OD_600_) was determined at a wavelength of 600 nm via a Genesys 20 photometer (Thermo Scientific GmbH, Dreieich, Germany). The culture broth was diluted with a 9 g NaCl L^−1^ solution to values between 0.1 and 0.3 before the measurement.

### Sulfite system for the determination of the maximum oxygen transfer capacity

A 0.5 M Na_2_SO_3_ solution was buffered by a 12 mM phosphate buffer. The phosphate buffer consisted of 0.5 mol L^−1^ Na_2_HPO_4_·2 H_2_O and 0.5 mol L^−1^ NaH_2_PO_4_·2 H_2_O. The pH of the final solution was adjusted to pH 8 with 30% w/w H_2_SO_4_. Afterwards, the solution is gassed with nitrogen. Under nitrogen aeration, 10^–7^ M CoSO_4_·7 H_2_O was added (Hermann et al. 2001). 25 mL of the solution was filled in the large perforated ring flask (Fig. 2). The flask was incubated at 200, 250 and 300 rpm at 25 °C with a shaking diameter (d_0_) of 50 mm. With an in-house built RAMOS device, the OTR was measured.

### Stoichiometric calculations based on the total oxygen consumption

To calculate, how much oxygen was consumed, the OTR was integrated. The integral of the OTR reflects the total oxygen consumption (TOC) of the cultivation (Müller et al. 2018; Kolbeck et al. 2019). Stoichiometric calculations (Eq. 1) were performed (Philip et al. 2018) to derive the consumed glucose or the consumed ammonium from the TOC. Equation 1 describes the growth of *E. coli* on glucose:

1 $${\text{C}}_{{6}} {\text{H}}_{{{12}}} {\text{O}}_{{6}} + 0.{\text{57 NH}}_{{3}} + {2}.{\text{34 O}}_{{2}} \to {3}.{\text{38 CH}}_{{{1}.{7}}} {\text{O}}_{{0.{43}}} {\text{N}}_{{0.{17}}} + {2}.{\text{62 CO}}_{{2}} + {3}.{\text{99 H}}_{{2}} {\text{O}}$$

## Results and discussion

### Enabling online monitoring in large perforated ring flasks

In this study, perforated ring flasks larger than those introduced by Hansen et al. (2022) are presented. These flasks have a greater inner and outer diameter, compared to the flask introduced before. This greater diameter is necessary to implement the membrane-based fed-batch system. It also generates a higher gas/liquid mass transfer, even at elevated filling volumes. The flask (Fig. 2) has an outer diameter d_out_ of 95 mm with a wall thickness d_wall_ of 3.5 mm, resulting in an inner diameter of the outer compartment d_in_ of 88 mm. The concentric ring has an outer ring diameter d_ring,out_ of 85 mm with a wall thickness d_wall,ring_ of 2.5 mm, resulting in an inner diameter d_ring,in_ of 80 mm. A gap is created between the concentric, perforated inner ring and the outer cylinder with a gap length l_gap_ of 1.5 mm. The inner ring has two perforations which are 3 × 3 mm large.

To enable online monitoring of the respiration activity, an adapter (Fig. S2) was designed. The adapter was manufactured with a stereolithographic 3D printer Form 3 (Formlabs Inc, Somerville, USA). To apply the adapter in microbiology, to avoid contamination and to enable reuse of the adapter, the material must be autoclavable. The autoclavability was tested with tubes printed with the material “high temp” (Formlabs Inc, Somerville, USA). To assess the influence of autoclaving on the material, the tubes were autoclaved and the difference in length and shape were determined (Fig. S3). Since the effect of autoclavation on the tube shape was neglectable and the material remained intact, the material was used to manufacture the adapter (Fig. 2c, d). The adapter is screwed onto the ring flask. Within the adapter, plugs for air inlet and exhaust gas outlet are inserted (Fig. 2c). The plugs enable the connection to a RAMOS device and, thus, the monitoring of respiration activity. Additionally, a glass reservoir is screwed onto the adapter. Like in previous publications (Bähr et al. 2012; Philip et al. 2017, 2018; Habicher et al. 2019) a flexible silicone tube is attached to the bottom of the glass reservoir. At the lower end of the silicone tube a diffusion tip is mounted (Fig. S4). The diffusion tip rotates within the inner compartment of the large perforated ring flask and remains permanent immersed in the rotating bulk liquid, as indicated in Figs. 2c and S5. By rotating in phase with the liquid, the diffusion tip does not affect the hydrodynamics. The diffusion tip is equipped with a cellulose membrane and separates the culture broth and the feed solution. This membrane needs to be in permanent contact with the culture broth to enable diffusive mass transfer. Diffusive mass transfer through the membrane occurs if an appropriate concentration gradient of the fed component is adjusted between the feed solution in the reservoir and the culture broth (Bähr et al. 2012). The reservoir, which enables fed-batch cultivation, can be screwed off from the adapter. If a batch cultivation is conducted, the hole for the reservoir in the center of the adapter can be closed by a GL 18 lid. The online monitoring of the respiration activity of biological cultures is still possible.

### Batch cultivation in large perforated ring flasks

**Fig. 3:**
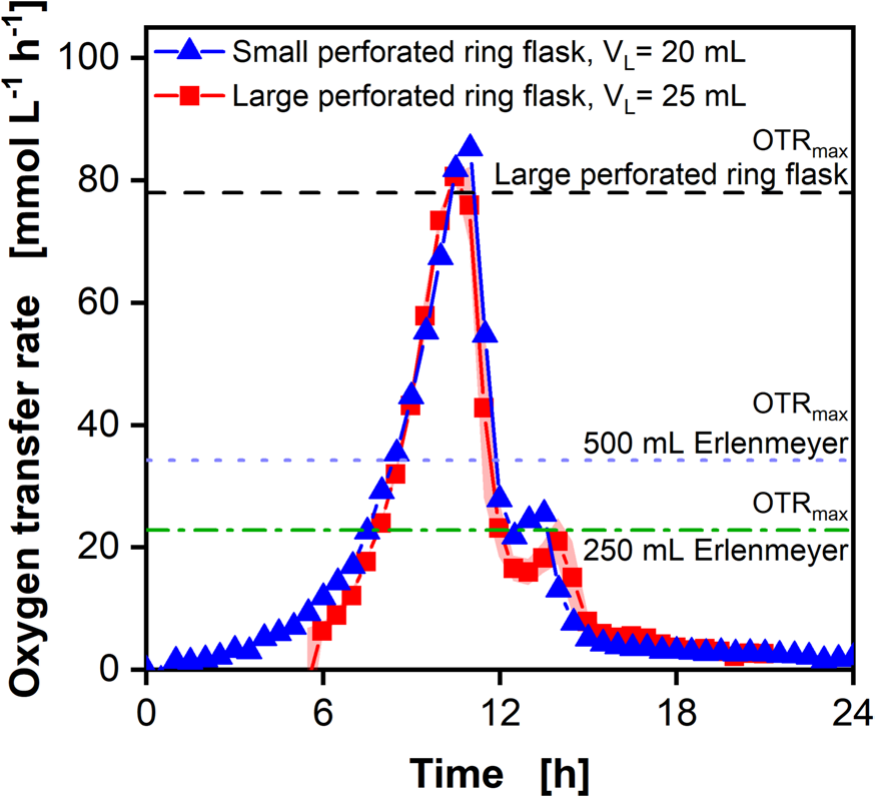
Comparison of the oxygen transfer rate (OTR) of *Escherichia coli* BL21 cultivations in two different perforated ring flasks in Wilms-MOPS mineral medium in batch mode. Experimental conditions for the large perforated ring flask: V_L_ = 25 mL, d_0_ = 50 mm, n = 250 rpm, T = 37 °C, initial optical density OD_600_ = 0.5, c_glucose_ = 20 g L^−1^, 200 mM MOPS buffer. For the cultivation in the large perforated ring flask, the mean value of two replicates was calculated and is represented by symbols and line. The minimum/maximum values are shown by a shadow behind the curve. The maximum oxygen transfer capacity (OTR_max_) was calculated according to Meier et al. (2016) for a 250 mL (horizontal green dash-dotted line) and a 500 mL (blue dotted line) Erlenmeyer flask. For the large perforated ring flask, a measurement applying a sulfite system was conducted, according to Hermann et al. (2001). Through the experimentally gained OTR_max_ values shown in Fig. S7 for 25 mL, the OTR_max_ for the large perforated ring flask was calculated with an osmolality of 0.68 mOsmol kg^−1^. For the flask dimensions, see Fig. 2. The shown OTR from the small perforated ring flask (blue triangle) is reproduced from Hansen et al. (2022), *E. coli* BL21, Wilms-MOPS medium, V_L_ = 20 mL, d_0_ = 50 mm, n = 250 rpm, T = 37 °C, initial optical density OD_600_ = 0.1. The OTR of the large perforated ring flask was adjusted in time by 5.5 h

To demonstrate the application of the online monitoring of the 3D-printed adapter (Fig. 2c, d), batch cultivations of *E. coli* BL21 in Wilms-MOPS medium were conducted in the large perforated ring flasks. Since only the online monitoring was validated, the diffusion tip was not yet attached to the adapter. Biocompatibility of the feed system was tested in a previous study (Fig. S6, Bähr et al. 2012). Figure 3 depicts the resulting oxygen transfer rate (OTR) in the large perforated ring flask with a filling volume of V_L_ = 25 mL, in comparison to the small perforated ring flask, introduced by Hansen et al. (2022), with a filling volume of V_L_ = 20 mL. The presented data for the cultivation with the small perforated ring flask are reproduced from Hansen et al. (2022). The OTR of the large perforated ring flask was adjusted in time by 5.5 h, since a higher initial optical density of OD_600_ = 0.5 was used in the large perforated ring flask, compared to an initial optical density of OD_600_ = 0.1 in the small perforated ring flask. The horizontal green dash-dotted line indicates the maximal oxygen transfer capacity (OTR_max_) calculated according to Meier et al. (2016) in a 250 mL shake flask and the blue dotted line in a 500 mL shake flask, considering a filling volume of V_L_ = 25 mL and a shaking frequency of n = 250 rpm. The black dashed line represents the OTR_max_ for the large perforated ring flask. It was evaluated by means of the sulfite system (Hermann et al. 2001, Fig. S7) and corrected for osmolality by the correlation of Meier et al. (2016). For the calculation, an osmolality of 0.68 mOsmol kg^−1^ for the Wilms-MOPS medium was applied.

The OTR of the cultivations in the large perforated ring flasks indicates a steep increase until it reaches a maximum OTR of 80.6 mmol L^−1^ h^−1^. This exceeds the OTR_max_ for the 250 mL Erlenmeyer flask by 3.5 times and the OTR_max_ for the 500 mL Erlenmeyer flask by almost 2.5 times. The OTR peak for cultivation in the large perforated ring flask is at the height of the calculated OTR_max_. At these elevated OTR values, the cultivation can move into an oxygen limitation (Meier et al. 2016). After the peak, a decrease in the OTR is visible, which indicates the total consumption of the initially present glucose (Anderlei and Büchs 2001; Philip et al. 2018). After 13 h, a second peak is visible in the OTR, where acetate is consumed, which was previously formed because of overflow metabolism (Anderlei and Büchs 2001; Phue and Shiloach 2005). Comparing the cultivations of the small perforated ring flask with the cultivations of the large perforated ring flask, the shape of the OTR curve is quite similar, even though a higher filling volume was used in the larger flask.

As a first step, the batch cultivations in the large perforated ring flasks validated, that the constructed adapter (Fig. 2c, d) without the feed reservoir can be applied in biological experiments and the respiration activity can be online measured during cultivation processes.

### Fed-batch cultivations in large perforated ring flasks

**Fig. 4:**
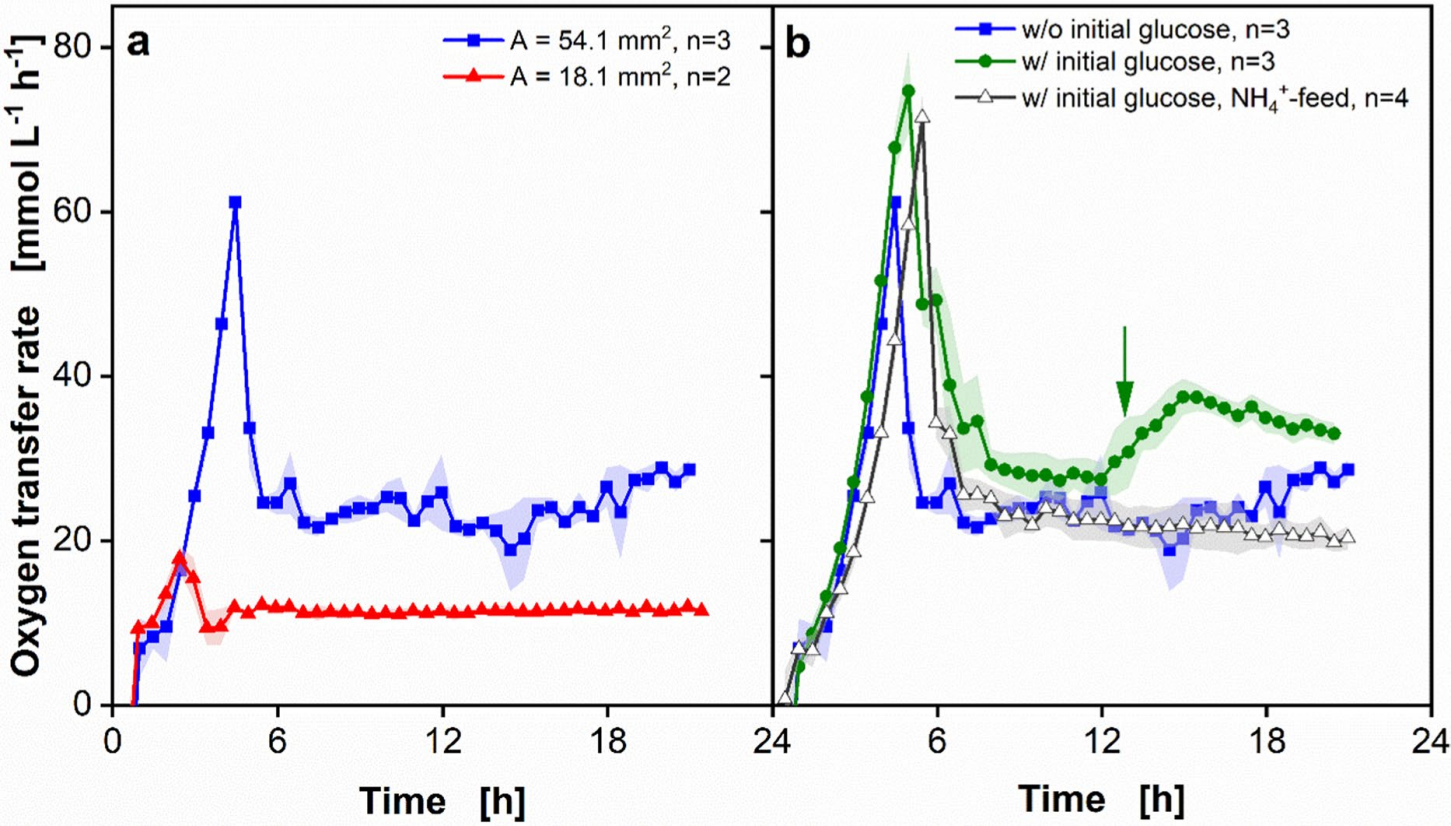
Comparison of oxygen transfer rates (OTR) of *Escherichia coli* BL21 fed-batch cultivations with diffusion tips with different diffusion areas and feed solutions in the large perforated ring flask. **a** OTR of cultivations with two different diffusion tips, a diffusion area of A = 18.1 mm^2^ (red triangle) and A = 54.1 mm^2^ (blue square). **b** OTR of cultivations with different initial glucose concentrations and with and without ammonium feed. w/o initial glucose represents experiments without initial glucose in the medium (data reproduced from **a**), w/ glucose represents experiments with 4 g glucose L^−1^ in the initial medium. In the cultivation with ammonium feed, 40 g ammonium L^−1^ was present in the reservoir. The diffusion area in **b** was 54.1 mm^2^. The green arrow indicates the time point, where the initial ammonium is in total consumed for the cultivation with glucose in the initial medium and without ammonium feed (green dot, see Fig. S8). The mean value from several experiments was calculated, which is shown as symbols and line. The standard deviation for n = 3 and n = 4 parallel experiments and the minimum/maximum value for n = 2 was calculated and is shown by a shadow behind the curve. Experimental conditions: V_L_ = 25 mL, d_0_ = 50 mm, n = 250 rpm, T = 37 °C, initial optical density OD_600_ = 0.5, V_reservoir_ = 3 mL, c_reservoir_ = 500 g L^−1^, Membrane type = RCT‐NatureFlex‐NP, material = regenerated cellulose, thickness d = 42 μm, cut‐off = 10–20 kDa

The application of the 3D-printed adapter with the feed reservoir in a fed-batch process was tested with *E.* *coli* BL21 cultivations (Fig. 4). The influence of the glucose release on cultivations in a fed-batch process was tested by using different diffusion tips (Fig. 4a), which differ in their diffusion area (Fig. S4b, c) and, consequently, in their glucose release. The diffusion area differs from A = 18.1 mm^2^ (red triangles) and A = 54.1 mm^2^ (blue squares). No glucose was initially present in the media. The reservoir feed solution contained of 500 g glucose L^−1^. The OTR displayed in Fig. 4a can be divided into two parts independent of the diffusion area. First, an increase in the OTR is visible and a peak is reached. This indicates the batch phase, where the glucose release rate is higher than the consumption rate of the organism. After the peak and the following decrease, a plateau in the OTR is visible. The plateau indicates the fed-batch phase, where the release rate is lower than the maximum consumption rate of the organisms. Thus, carbon-limited conditions are present (Jeude et al. 2006; Philip et al. 2017; Habicher et al. 2019). The OTR values of the cultivations with the tips with a diffusion area of A = 18.1 mm^2^ are around 18 mmol L^−1^ h^−1^ for the batch peak and around 11.7 mmol L^−1^ h^−1^ in the fed-batch phase. Compared to that, the OTR for the cultivations with the diffusion tip with a diffusion area of A = 54.1 mm^2^ is 61.2 ± 0.7 mmol L^−1^ h^−1^ in the batch peak and 24 ± 2.4 mmol L^−1^ h^−1^ in the fed-batch phase. Although the diffusion area is three times larger, the OTR in the fed-batch plateau is only two times higher. The same glucose concentration in the reservoir was used in both experiments, but the higher batch peak of the cultivation with the diffusion tip with a larger diffusion area indicates that more glucose was already consumed in the batch phase, compared to the cultivation with the smaller diffusion tip. To prove this, stoichiometric calculations were conducted considering Eq. (1). Glucose concentrations in the batch peak of 1.3 ± 0.1 g L^−1^ for diffusion tips with A = 18.1 mm^2^ were calculated. For diffusion tips with A = 54.1 mm^2^, glucose concentrations in the batch peak of 6.3 ± 0.2 g L^−1^ were calculated. This leads to a reduction of the glucose amount left in the reservoir. Thus, a lower concentration gradient over the membrane is present, which consequently results in a reduced driving force for glucose diffusion (Philip et al. 2017).

Comparing Figs. 3 and 4a, it is obvious that the OTR peak in batch cultivations is higher than in the fed-batch cultivations. To achieve a higher batch peak, glucose can initially be added to the medium. The determined OTR of cultivations with a reservoir concentration of 500 g glucose L^−1^ and an initial glucose concentration of 4 g L^−1^ is displayed in Fig. 4b (green dots). The OTR initially increases to an OTR value of 74 ± 4.5 mmol L^−1^ h^−1^. Hence, with the additional initial glucose, a higher batch peak and almost the calculated OTR_max_ (Fig. 3) for the large perforated ring flask was reached. A second slow increase in the OTR of the cultivations with initial glucose is visible after 13 h (Fig. 4b, green arrow). This increase indicates an ammonium limitation (Philip et al. 2018). To prove this hypothesis, stoichiometric calculations with Eq. (1) were conducted (Fig. S8, Philip et al. 2018). It is depicted in Fig. S8 that ammonium is depleted at the time point, where the slow increase in the OTR is visible. To avoid an ammonium limitation, addition of ammonium in the initial medium or the feed reservoir is possible. Philip et al. (2018) showed that with increasing initial ammonium concentration in the medium the increase in the OTR plateau is delayed, the lag phase is significantly prolonged and the growth rate decreases. Furthermore, oxygen limitations occurred during the batch phase. Adding ammonium to the reservoir, the increase in the OTR plateau is delayed as well, the growth rate only decreased slightly and no oxygen limitation occurred (Philip et al. 2018). Therefore, in this study, ammonium was added to the feed reservoir solution instead of the culture medium. The OTR of the cultivations with initial glucose in the medium and an ammonium feed is shown in Fig. 4b (black triangles). The OTR increases until a batch peak of 71 ± 2.9 mmol L^−1^ h^−1^ is reached. The increase in the OTR at the beginning of the cultivations with ammonium feed and initial glucose is slightly slower than the increase of the other two conditions. To prove this, the maximum growth rate until the batch peak is reached was calculated (Stöckmann et al. 2003). The exponential increase was determined with help of an exponential trend line, which was created through at least six data points and had an R^2^ = 0.99. The growth rate decreases from µ_max,batch_ = 0.71 ± 0.05 h^−1^ for the cultivations with initial glucose to µ_max,batch_ = 0.56 ± 0.02 h^−1^ in the cultivations with initial glucose and ammonium feed. A reason for this observed phenomenon could be a higher osmolality at the beginning of the cultivation when glucose and ammonium diffuse into the culture broth (Philip et al. 2018). The OTR of the cultivation with ammonium in the feed reservoir does not increase during the plateau. Thus, the ammonium limitation can be prevented by adding ammonium to the feed solution. Furthermore, the final OD_600_ was measured to investigate whether the ammonium feed enhances the biomass formation. By adding ammonium to the glucose feed, the optical density increases from 27 ± 0.1 without ammonium feed to 31.1 ± 1.3 with additional ammonium feed. These results demonstrate the positive effect of the ammonium addition to the glucose feed and underlines that an ammonium limitation was prevented.

During cultivation, a formation of foam was observed in the large perforated ring flask (Fig. S9a). An influence of the diffusion tip on foam formation can be excluded, since the formation of foam was also observed in batch mode without the diffusion tip (Fig. S9b, c). It is obvious that even without the diffusion tip heavy foam is generated. Therefore, the diffusion tip is not the reason for the foam formation. Furthermore, the bulk liquid in the outer compartment showed an angular shift relative to the liquid in the inner compartment. To exclude that this phenomenon only occurs, when biological cultivations are conducted, an abiotic fed-batch process with colored water was performed (Fig. S10). Even with water, the bulk liquids in the two compartments were angularly shifted relatively to each other and small bubbles appeared. An explanation for this phenomenon could be the small gap size l_gap_ of 1.5 mm (Fig. 2a). The gap could act as a capillary so that the liquid rises to a higher level in the gap than in the inner compartment. The assumption that the capillary effect between the two cylinders of the perforated ring flask on the rotating liquid hinders mixing was discarded since mixing times of 5 s were observed (Fig. S11). The relation between the capillary effect and the heavy foaming should be investigated in further studies. Another challenge could be the cultivation of filamentous or aggregating microorganisms in the perforated ring flask since aggregates could clog the perforation. For single cellular organisms like *E. coli* clogging of the perforation, however, is not an issue. Therefore, the large perforated ring flask can be regarded as a useful cultivation tool for unicellular microorganisms.

Looking at Fig. 4, it is striking that only a small standard deviation (depicted as shadows) can be observed. Therefore, despite of the foaming, very reproducible results were obtained. This should be emphasized because not only the biology, but also the online measurement technology and the diffusive mass transfer of glucose (and ammonium) through the membrane can deviate and can influence the reproducibility. In addition, the perforated ring flasks are tailor-made and, therefore, may have small geometric variations.

## Conclusion

In this study, a new and large perforated ring flask with increased gas/liquid mass transfer was introduced. To enable online monitoring of biological cultures with a RAMOS device and to enable batch and fed-batch cultivations, a 3D-printed adapter was designed and manufactured by stereolithographic 3D-printing. At the same operating conditions, batch cultivations show 3.5 times higher maximum oxygen transfer capacity compared to Erlenmeyer flasks. Even with commercially available shakers and practical operating conditions of 25 mL filling volume and a shaking frequency of 250 rpm, OTR_max_ values of 80 mmol L^−1^ h^−1^ were achieved.

The establishment of a membrane-based fed-batch operation in the large perforated ring flask was investigated. Oxygen transfer rates during the fed-batch phase of 24 mmol L^−1^ h^−1^ could be achieved by applying diffusion tips with a diffusion area of 54.1 mm^2^. These high oxygen transfer rates cannot be reached in standard Erlenmeyer flasks with elevated filling volumes and low shaking frequencies. Since processes show a higher reproducibility if their pre-cultures are grown under fed-batch conditions (Keil et al. 2019), the large perforated membrane-based ring flask can be useful, when high amounts of pre-culture volume are required. Furthermore, since the main culture of industrial processes is often performed under fed-batch conditions, the membrane-based perforated ring flask can also be a useful tool for screening and process development of biotechnological processes. Furthermore, secondary substrate limitations could be observed through online monitoring the OTR. By applying a feed solution with 40 g ammonium L^−1^ in the reservoir, this secondary substrate limitation could be prevented.

During cultivation in batch as well as in fed-batch mode, foam was generated. In addition, the bulk liquid in the two compartments showed an angular shift relatively to each other. But, even in spite of the observed formation of foam, highly reproducible results were obtained. This is remarkable considering that not only the biology, but also the online monitoring, the diffusive mass transfer through the membrane and small geometric variations of the tailor-made large perforated ring flasks influences the reproducibility.

In this study, the combination of three established technologies, the membrane-based feeding in shake flasks (Bähr et al. 2012; Philip et al. 2017), the RAMOS device (Anderlei et al. 2004), and the newly introduced perforated ring flask (Hansen et al. 2022), was successfully applied. This combination represents a large potential to improve screening and process development in biotechnology processes.

### Supplementary Information

Below is the link to the electronic supplementary material.

Supplementary file1 (PDF 1654 kb)
