# Supplementary material for: Diffusion-driven fed-batch fermentation in perforated ring flasks
Source: Biotechnol Lett. 2024 May 17;46(4):571–82. doi: 10.1007/s10529-024-03493-0 (PMC11217090; doi:10.1007/s10529-024-03493-0)
Supplement: Supplementary file 1 — Supplementary file1 (PDF 1654 kb) [file 10529_2024_3493_MOESM1_ESM.pdf]

Supplementary Data

Diffusion-driven fed-batch fermentation in perforated ring flasks

Clara Lüchtrath[1], Felix Lamping[1], Sven Hansen[2], Maurice Finger[1], Jørgen Magnus[1], Jochen Büchs[1]*

[1]AVT – Biochemical Engineering, RWTH Aachen University, Forckenbeckstraße 51, 52074 Aachen, Germany

[2]Evonik Operations GmbH, Paul-Baumann-Straße 1, 45772 Marl, Germany

[*]Correspondence: jochen.buechs@avt.rwth-aachen.de

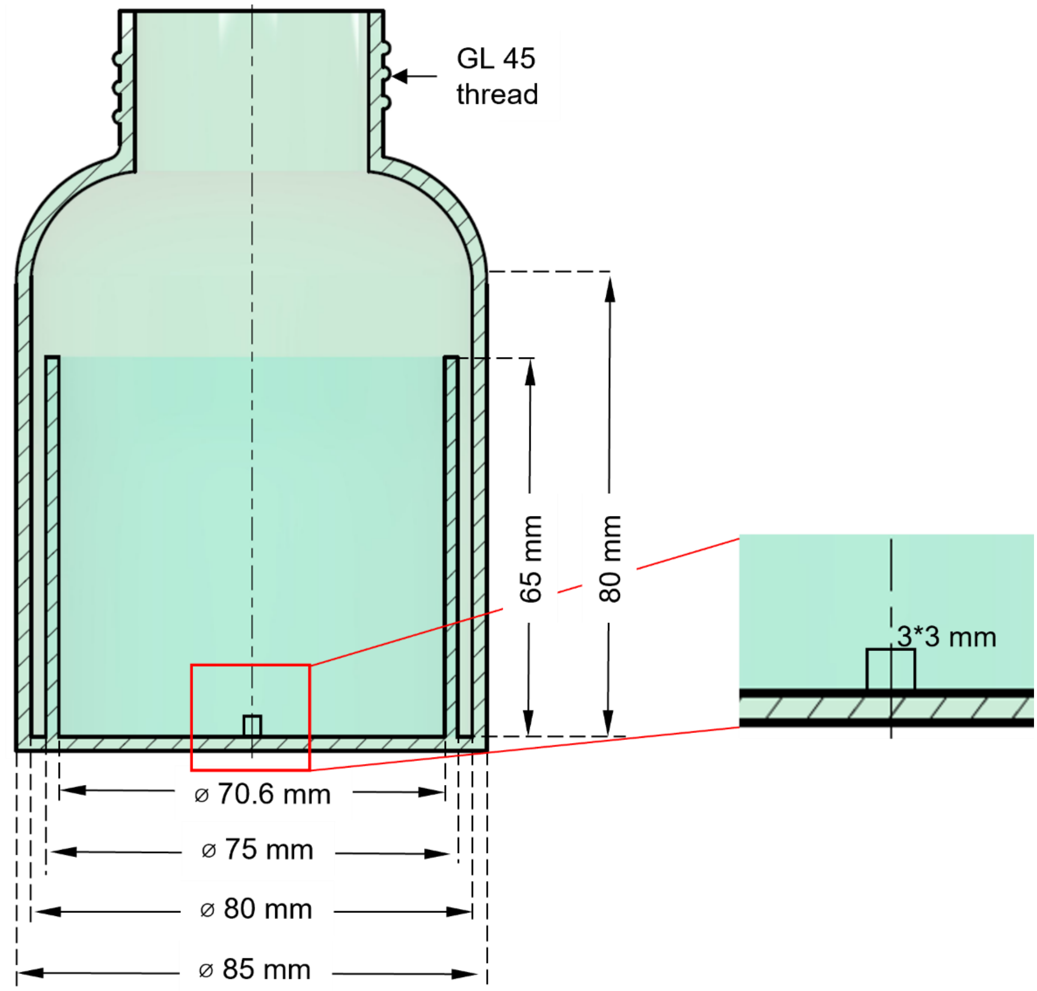

Fig. S 1 Schematic representation of the small perforated ring flask. The inner ring contains two perforations (3 x 3 mm). Flask dimensions according to Hansen et al., 2022. The close-up view shows the perforation of the inner ring, which is 3 * 3 mm in size

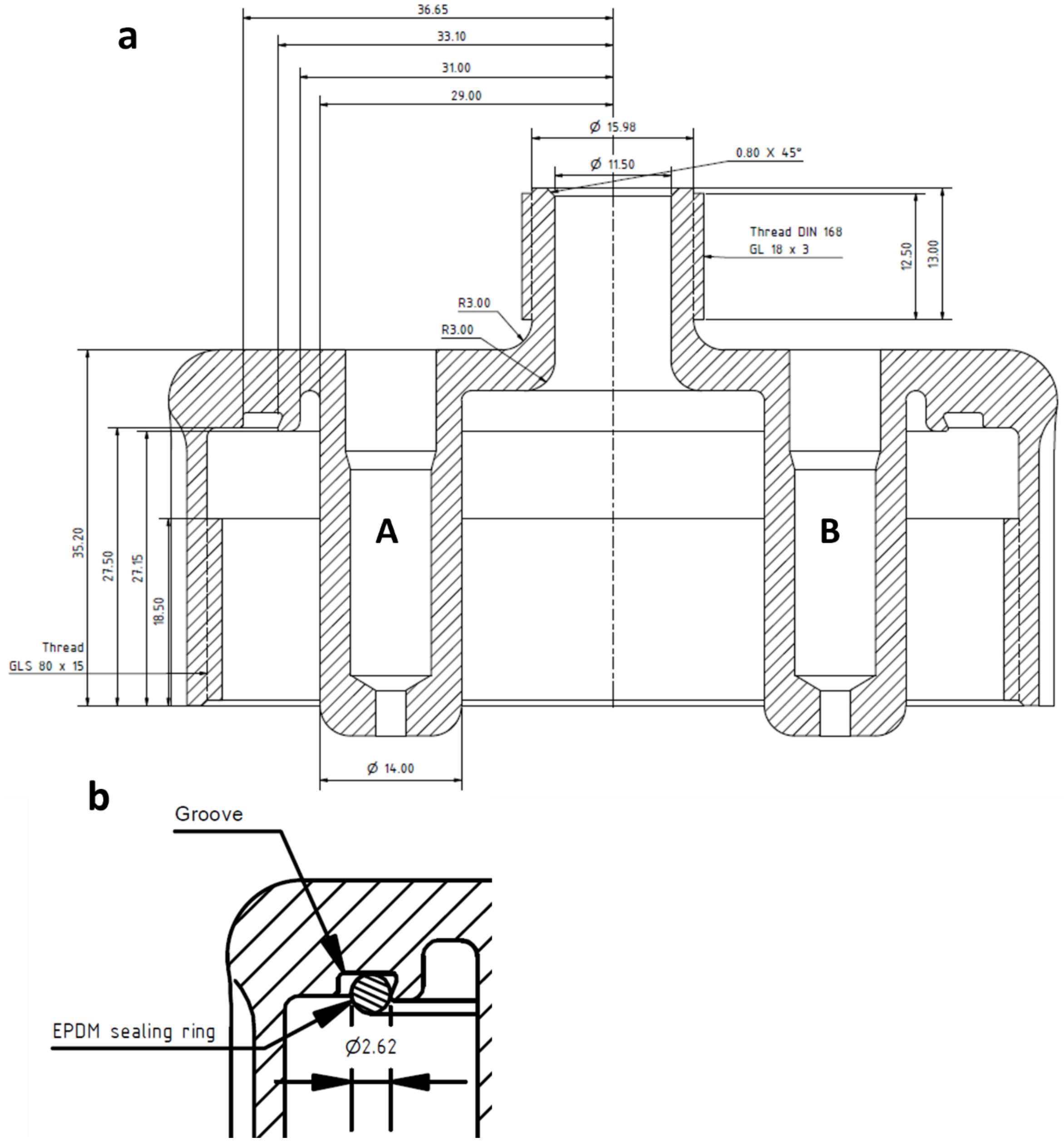

Fig. S 2 Schematic representation of the 3D-printed adapter enabling respiration activity monitoring with a RAMOS device. (a) Section through the adapter with holes for the plugs connected to air inlet (A) and exhaust gas outlet (B). (b) Magnified view of the groove and the ethylene propylene diene monomer (EPDM) rubber sealing ring

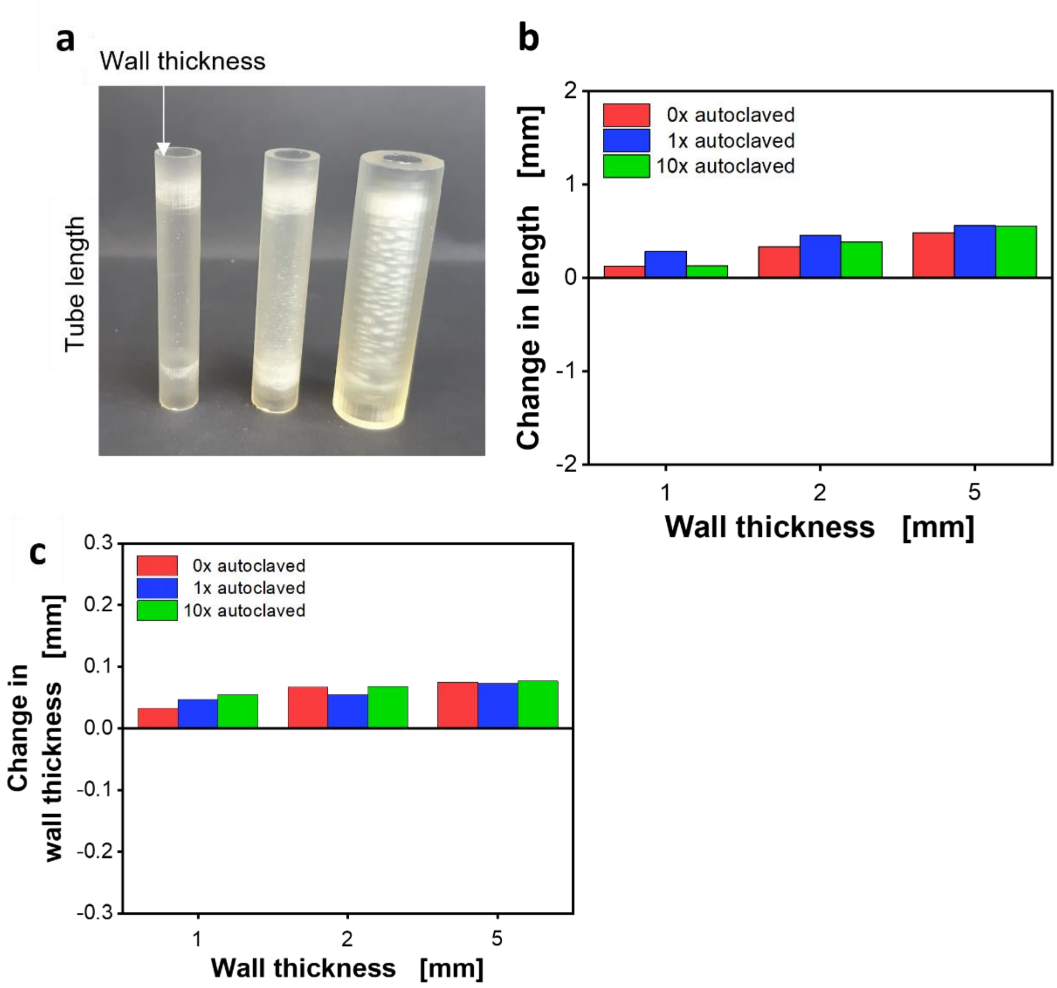

Fig. S 3 Evaluation of the shape retention of the 3D-printed material. (a) Test tubes produced by 3D-printing (stereolithography) with different thicknesses of 1, 2 and 5 mm. (b) Change in length of the test tubes after 0, 1 or 10 autoclavation cycles. (c) Change in wall thickness of the test tubes after 0, 1 or 10 autoclavation cycles

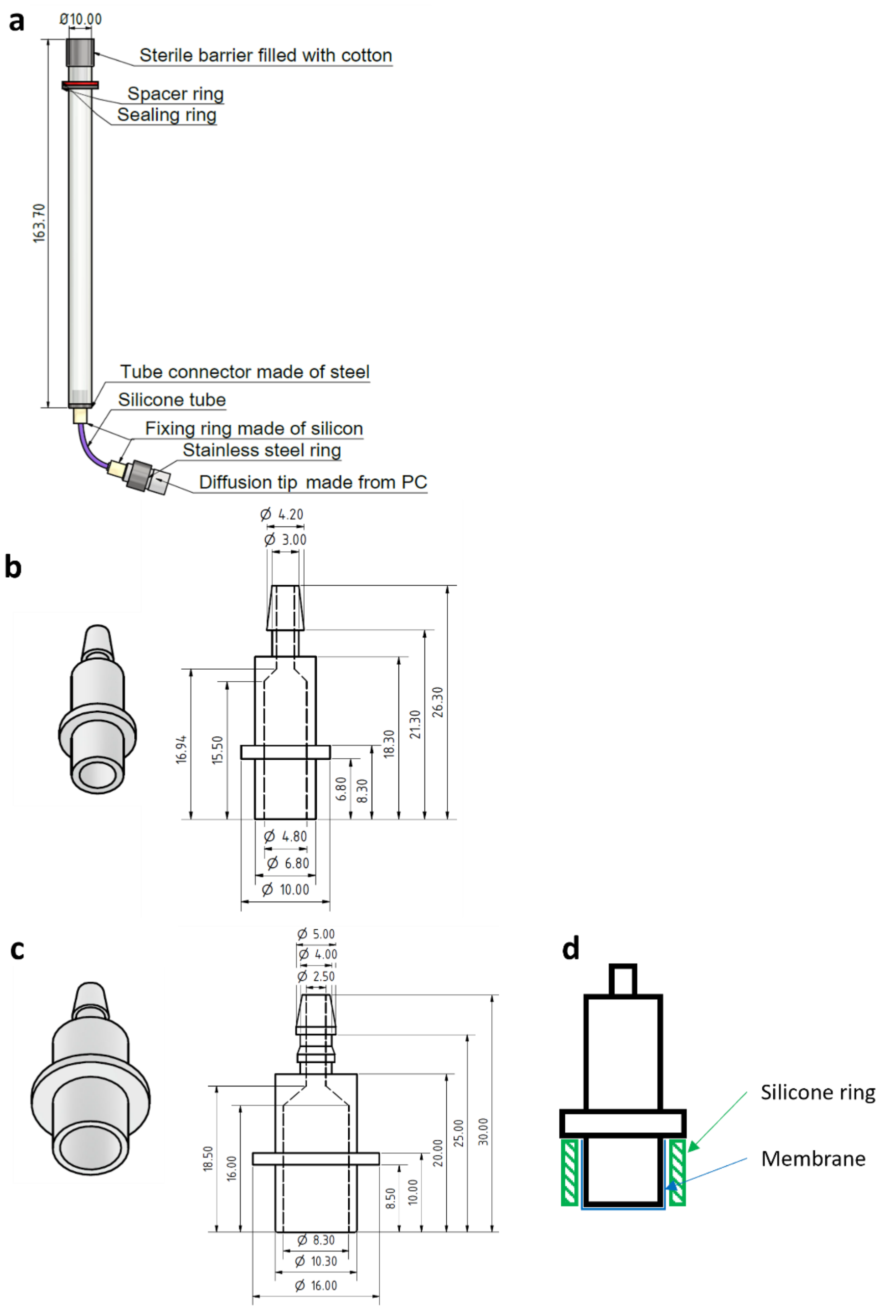

Fig. S 4 Illustration of the feed reservoir and the diffusion tips for the membrane-based fed-batch in ring flasks. (a) Illustration of the feed reservoir for diffusive mass transfer of nutrients into the cultivation broth for the perforated ring flask with an assembled diffusion tip made from polycarbonate (PC). The glass tube (Schott Duran®) used as a reservoir has an outer diameter d_{out} of 10 mm, and an inner diameter d_{in} of 7 mm. (b) + (c) Different diffusion tips that differ in their diffusion area with A = 18.1 mm^2 (d_{tip1} = 4.8 mm) (b) and A = 54.1 mm^2 (d_{tip2} = 8.3 mm) (c). (d) Illustration of a diffusion tip equipped with a cellulose membrane. The cellulose membrane is held in place with a silicone ring

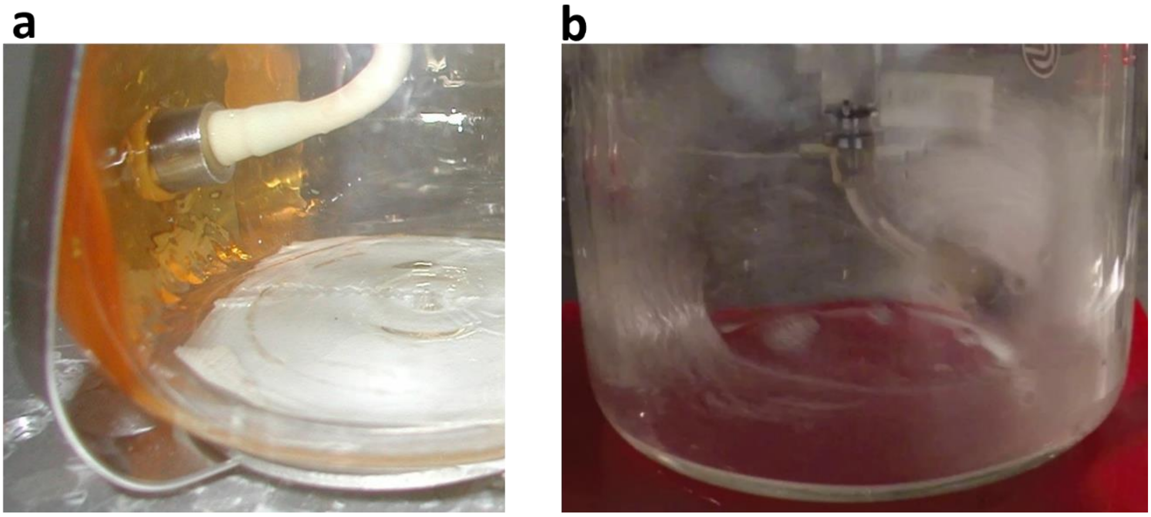

Fig. S 5 Contact of the diffusion tip with the rotating bulk liquid (a) in a 250 mL shake flask at a shaking frequency of 350 rpm and (b) in the inner compartment of the large perforated ring flask. The picture in (b) was taken during a fed-batch cultivation of *E. coli* with a shaking frequency of n = 250 rpm and a shaking diameter of d_0 = 50 mm

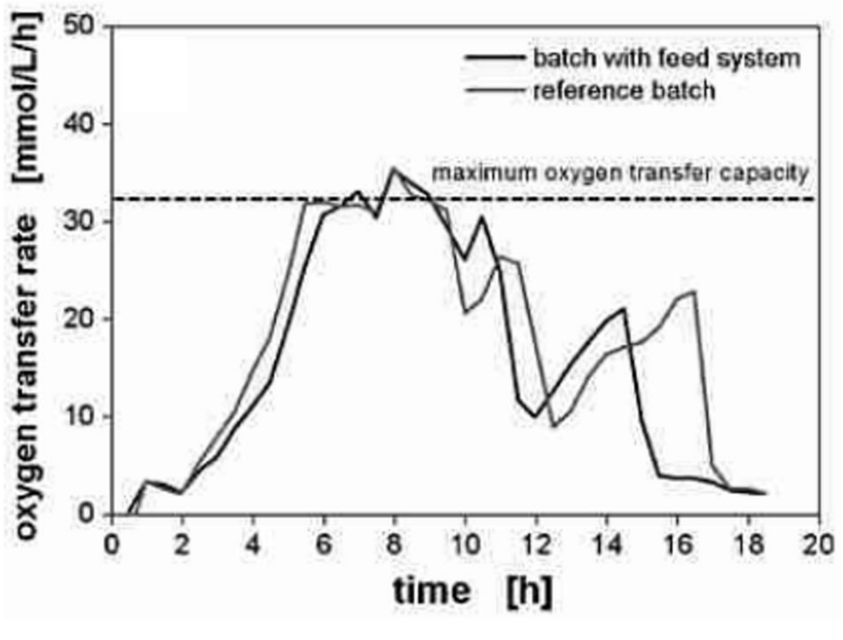

Fig. S 6 Biocompatibility of the membrane-based fed-batch shake flask with a fully equipped feed system, but an empty feed reservoir. The figure is adapted and reproduced from Bähr et al. (2012) with permission of the corresponding author

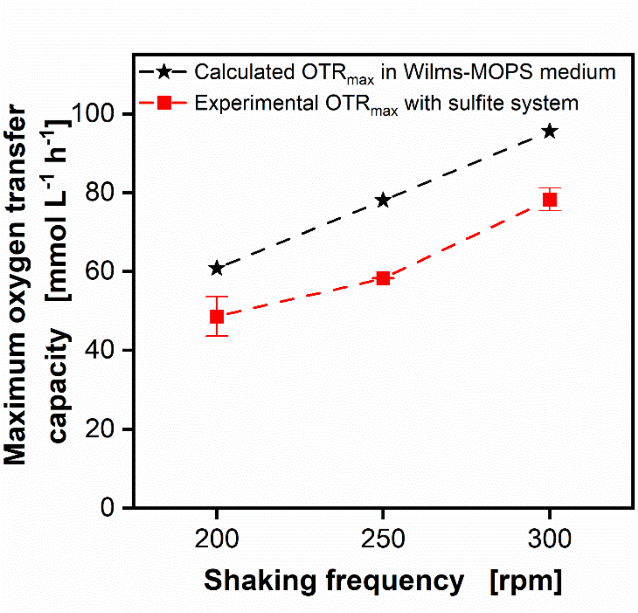

Fig. S 7 Maximum oxygen transfer capacity (OTR_{max}) of the large perforated ring flask**.** The OTR_{max} of the large perforated ring flask was determined using a 0.5 M sulfite system (1.18 mOsmol kg^{-1}, red squares), according to Hermann et al. (2001). The calculated OTR_{max} for the large perforated ring flask are based on the experimental OTR_{max} and were calculated according to (Meier et al. 2016) with the osmolality for the Wilms-MOPS-media (0.68 mOsmol kg^{-1}, black stars). The conditions for the large perforated ring flask experiment were: V_L = 25 mL, d_0 = 50 mm, T = 25 °C. For flask dimension, see Fig. 2

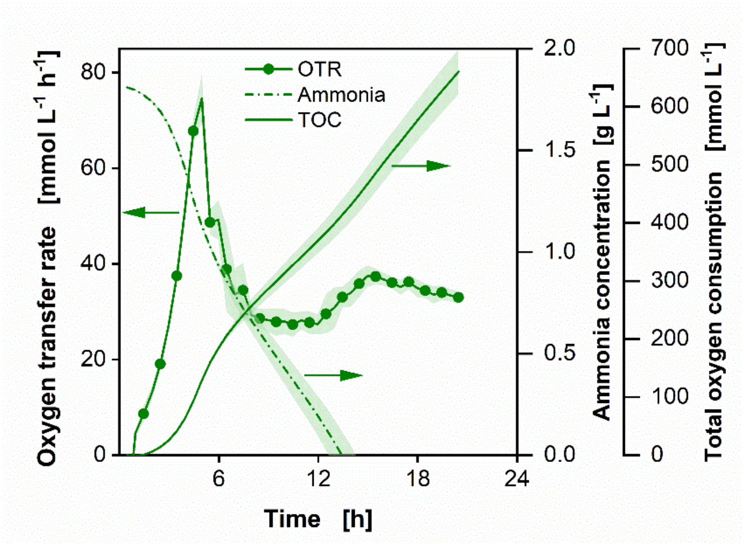

Fig. S 8 Oxygen transfer rate and ammonia concentration of an *Escherichia coli* BL21 fed-batch cultivation in the large perforated ring flask**.** OTR of a cultivation with 4 g glucose L^{-1} in the initial medium correspond to Fig. 3b, green dots. In the feed reservoir, 500 g glucose L^{-1} was present. For clarity, only every third measurement value is shown as a symbol. For n = 3 replicates, the mean value is shown. The shadows indicate the standard deviation. Experimental conditions: V_L = 25 mL, d_0 = 50 mm, n = 250 rpm, T = 37 °C, initial optical density OD_{600} = 0.5, $V_{reservoir}$ = 3 mL, Membrane type=RCT-NatureFlex-NP, material = regenerated cellulose, d = 42µm, cut-off = 10–20 kDa. For flask dimension, see Fig. 2. For calculations of the ammonia consumption (dot dashed curve), the stoichiometric equation (1) was used. Calculations are based on the total oxygen consumption (TOC, line), which can be determined by the integral of the OTR

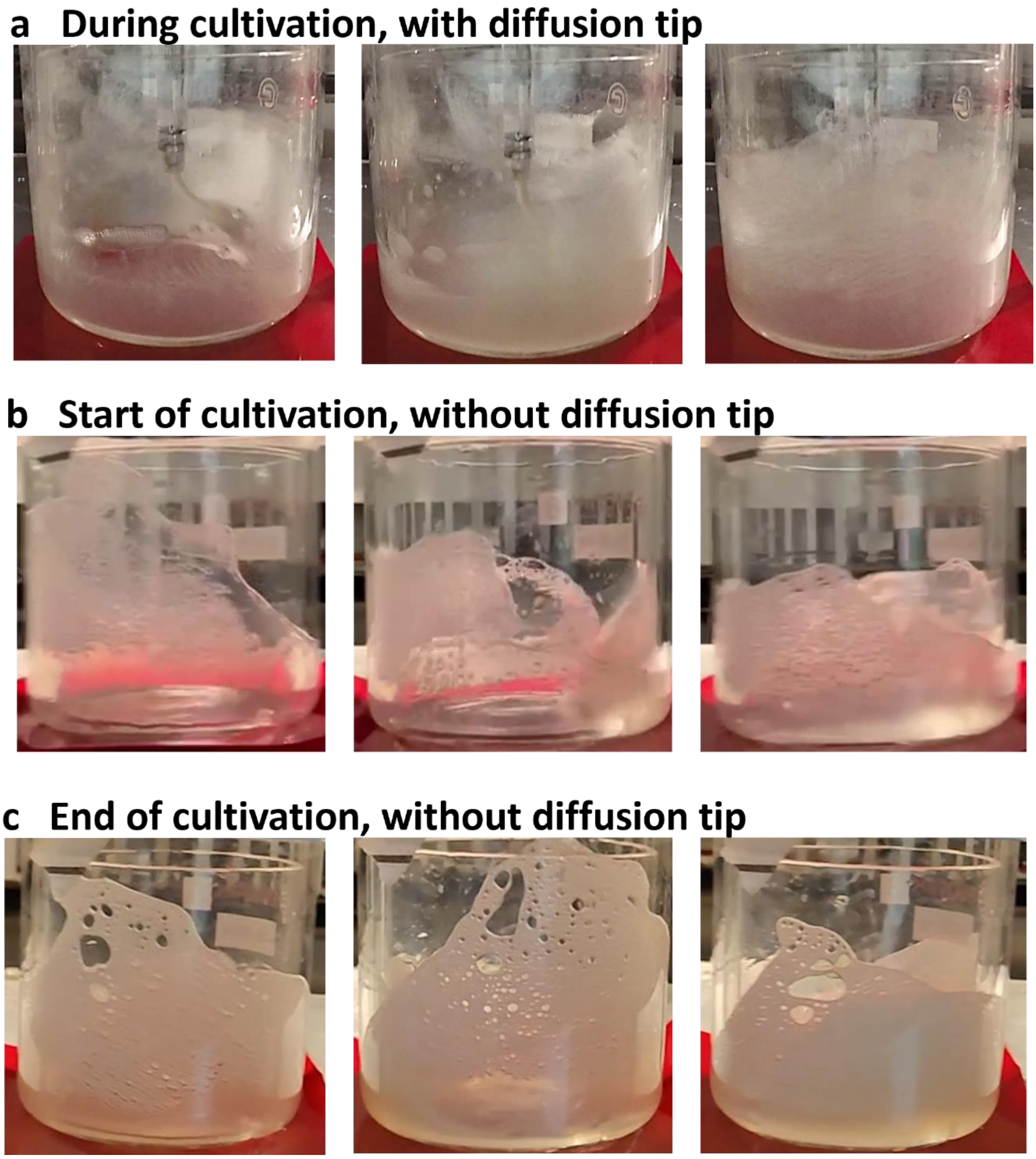

Fig. S 9 Foam formation in the inner and outer compartment of the large perforated ring flask. (a) During cultivation in fed-batch mode with an assembled feeding system with diffusion tip. (b) At the beginning of the cultivation in batch mode without a feeding system. (c) At the end of the cultivation after 20 h in batch mode. Three exemplary pictures for all conditions are shown. Cultivation condition: *E. coli* BL21 in Wilms-MOPS medium, V_L = 25 mL, d_0 = 50 mm, n = 250 rpm, T = 37 °C, initial optical density OD_{600} = 0.5. For flask dimension, see Fig. 2

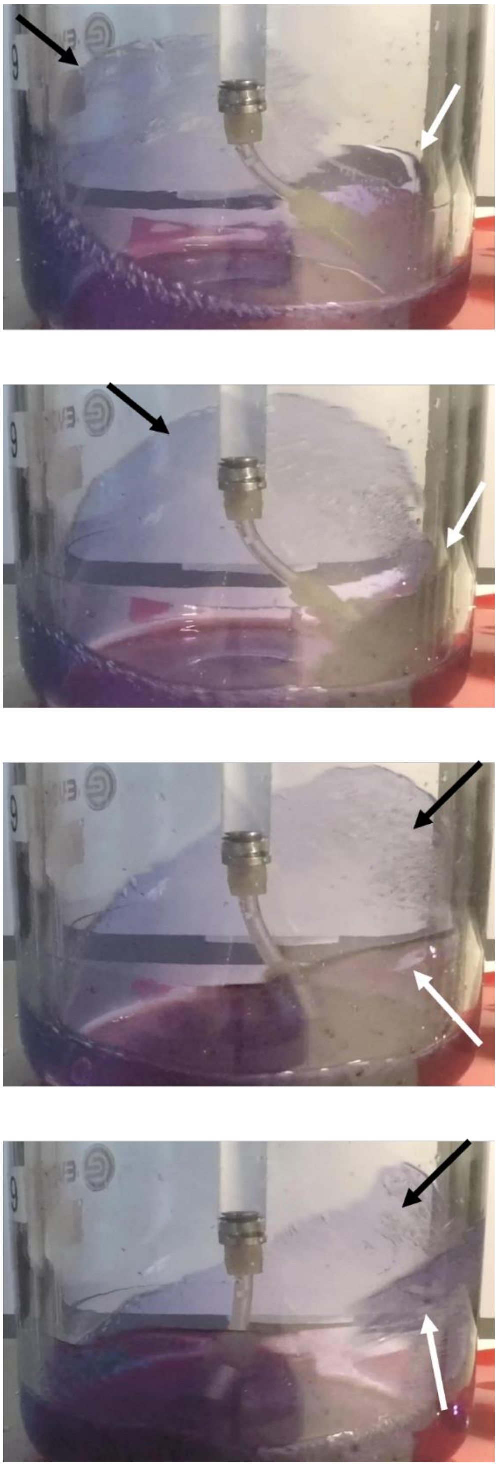

Fig. S 10 Liquid distribution in the large perforated ring flask mimicking fed-batch mode. Abiotic experiment with water colored with ink. Four exemplary pictures are presented. The white arrow points at the inner bulk liquid, the black arrow point at the outer bulk liquid. Condition: V_L = 25 mL, d_0 = 50 mm, n = 250 rpm. For flask dimension, see Fig. 2

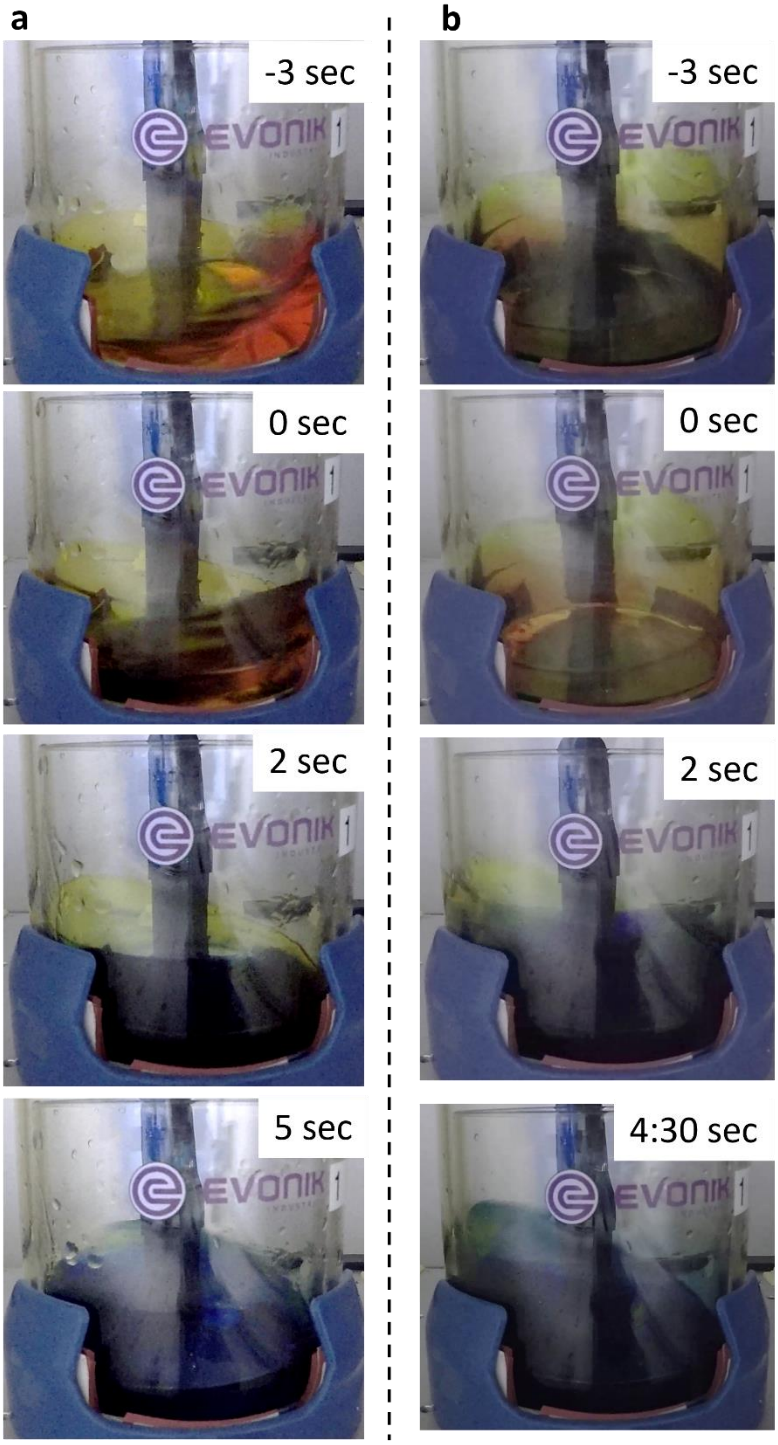

Fig. S 11 Determination of the mixing time by color change using a pH indicator. Investigation of the mixing behavior according to Tan et al. (2011) at (a) 100 rpm and (b) 190 rpm. 0 seconds marks the time point of adding the pH adjusting agent. Thymol blue was used as the pH indicator. The pH adjusting agent, to achieve the color change from yellow to blue, was NaOH

References

Bähr C, Leuchtle B, Lehmann C, Becker J, Jeude M, Peinemann F, Arbter R, Büchs J (2012) Dialysis shake flask for effective screening in fed-batch mode. Biochem Eng J 69:182–195. https://doi.org/10.1016/j.bej.2012.08.012

Hermann R, Walther N, Maier U, Büchs J (2001) Optical method for the determination of the oxygen-transfer capacity of small bioreactors on sulfite oxidation. Biotechnol Bioeng. https://doi.org/10.1002/bit.1126.

Meier K, Klöckner W, Bonhage B, Antonov E, Regestein L, Büchs J (2016) Correlation for the maximum oxygen transfer capacity in shake flasks for a wide range of operating conditions and for different culture media. Biochem Eng J 109:228–235. https://doi.org/10.1016/j.bej.2016.01.014

Tan R-K, Eberhard W, Büchs J (2011) Measurement and characterization of mixing time in shake flasks. Chemical Engineering Science 66:440–447. https://doi.org/10.1016/j.ces.2010.11.001
